# Enhanced Antioxidant and Protective Effects of Fermented *Solanum melongena* L. Peel Extracts Against Ultraviolet B-Induced Skin Damage

**DOI:** 10.3390/nu17050847

**Published:** 2025-02-28

**Authors:** Joo Hwa Lee, Jinsick Kim, Yu Chang Jo, Yun Hoo Jo, Yeong Hwan Jeong, Soo Ah Jeong, Beong Ou Lim, Dong Wook Shin

**Affiliations:** 1 Research Institute for Biomedical and Health Science, Konkuk University, Chungju 27478, Republic of Korea; hesiit@kku.ac.kr (J.H.L.); jindoli477@kku.ac.kr (J.K.); julie3734@kku.ac.kr (Y.H.J.); 2 Department of Applied Biochemistry, Konkuk University, Chungju 27478, Republic of Korea; reeds1996@kku.ac.kr (Y.C.J.); mijo6064@kku.ac.kr (Y.H.J.); jje318@kku.ac.kr (S.A.J.); 3 Human Bioscience Corporate R&D Center, Human Bioscience Corp., 268 Chungwondaero, Chungju 27478, Republic of Korea

**Keywords:** eggplant peels, bio-conversion, human dermal fibroblasts, hydrogen peroxide, ultraviolet B, skin aging

## Abstract

**Background/Objectives**: The skin, being the body’s outermost organ, plays a vital role in protecting against various external stimuli. Ultraviolet generates reactive oxygen species (ROS), promoting the secretion of matrix metalloproteinases (MMPs) and inducing collagen degradation. Many studies have been conducted to identify natural substances that can prevent or delay the harmful effects of UV. **Methods**: A wound healing assay, DCF-DA reactive oxygen species (ROS) assay, and JC-1 assay were performed to assess the effects of bio-converted eggplant peels (BEPs) on human dermal fibroblasts (HDFs). Western blot analysis was also conducted to understand the underlying mechanisms for their effects. Finally, hematoxylin–eosin staining and immunohistochemistry were also performed in animal studies. **Results**: Our study evaluated the antioxidant efficacy of BEPs fermented with *Lactobacillus plantarum* in hydrogen peroxide (H_2_O_2_)-HDFs and UVB-induced skin damage in hairless mice. We demonstrated that BEPs exhibited enhanced antioxidant properties compared to non-fermented eggplant peels (EPs). BEPs facilitated wound healing in H_2_O_2_-damaged HDFs, reduced ROS levels, and restored mitochondrial membrane potential. BEPs suppressed the phosphorylation of ERK, p38, and JNK as their underlying mechanism. We further demonstrated that dietary supplementation of BEPs also downregulated matrix metalloproteinase 1 (MMP1) expression and upregulated collagen I (COL1) in UVB-damaged hairless mice, indicating that BEPs were more effective compared to EPs. **Conclusions**: Our studies suggest that BEPs fermented with *Lactobacillus plantarum* hold significant potential as a protective agent for mitigating UVB-induced damage and promoting skin health.

## 1. Introduction

The skin is constantly exposed to various external stimuli that influence its morphology and function [1]. It plays a vital role in safeguarding the body from external insults and preserving homeostasis [2]. Structurally, the skin consists of the epidermis, dermis, and subcutaneous tissue. The epidermis serves as a physical barrier, preventing water loss and protecting against environmental damage. The dermis contains fibroblasts and the extracellular matrix (ECM), which are essential for maintaining skin elasticity and strength [3,4,5]. The subcutaneous tissue, primarily composed of adipose cells, serves as a cushion and energy reservoir [6].

The skin is subjected to various external stressors, including ultraviolet (UV) radiation, pollutants, and chemical irritants. Ultraviolet (UV) radiation causes oxidative stress [7,8]. Excessive ROS production leads to oxidative damage, targeting proteins and DNA, and ultimately impairing cellular function and survival [9,10]. Beyond oxidative stress, chronic UV exposure causes skin carcinogenesis by inducing direct DNA damage. UV radiation generates 6-4 photoproducts (6-4PPs) and cyclobutane pyrimidine dimers (CPDs), which induce mutations in key regulatory genes. The severity of UV-induced damage depends on multiple factors, including age at exposure, frequency, duration, geographic location, and individual skin phototype [11,12,13].

Excessive ROS levels trigger the activation of the mitogen-activated protein kinase (MAPK) pathway, which promotes the expression of matrix metalloproteinases (MMPs) and accelerates collagen degradation, resulting in structural damage [14,15,16]. Moreover, ROS-induced mitochondrial dysfunction disrupts energy metabolism, exacerbating oxidative stress and further compromising skin health [17]. These processes collectively undermine the structural and functional integrity of the skin, resulting in visible signs of aging.

To counteract the detrimental effects of ROS, extensive research has focused on identifying natural antioxidants capable of mitigating oxidative stress. Plant-derived compounds, in particular, have demonstrated significant potential due to their potent antioxidant properties [18]. *Solanum melongena* L. (eggplant) reduces chronic degenerative diseases when included in the diet, primarily owing to the bioactive ingredients present in its peel [19,20,21]. Furthermore, the peel of eggplant is especially concentrated with these bioactive compounds, exhibiting potent antioxidant activity [22,23].

Probiotics are live microorganisms that, when taken in sufficient quantities, offer health advantages to the host and contribute to immune system regulation [24,25,26]. Recent investigations into probiotic microorganisms as natural antioxidants have shown promising results. *Lactobacillus*, a major constituent of probiotics, has demonstrated significant antioxidant potential in various studies on lactic acid bacteria [27,28,29,30]. Additionally, certain bacterial products with antioxidant activity have been identified as key players in skincare [31]. Recent studies highlighted the beneficial effects of *Lactobacillus plantarum* to alleviate symptoms of diabetes in human subjects [32,33] while exhibiting notable anti-inflammatory effects [34,35].

In this study, we selected *Lactobacillus plantarum* for the fermentation of eggplant peel extracts (BEPs) due to its well-documented antioxidant and anti-inflammatory properties. Previous studies have reported that fermentation using *L. plantarum* enhances the bioavailability of polyphenols and other bioactive compounds, potentially augmenting their protective effects against oxidative stress [36,37]. Thus, we hypothesized that fermenting eggplant peel with *L. plantarum* would enhance its antioxidant capacity and improve its protective effects against ROS-induced skin damage.

Based on these findings, this study evaluated whether eggplant peel extracts fermented with *Lactobacillus plantarum* (BEPs) could mitigate skin damage in human dermal fibroblasts (HDFs) caused by H_2_O_2_.

## 2. Materials and Methods

### 2.1. Preparation of Fermented Solanum melongena L. (Eggplant) Peel Extracts

*Solanum melongena* L. (eggplant) peel was dried at 40 °C in an oven for 3 days to remove moisture and then subjected to triple extraction with 70% ethanol at room temperature for 2 h each time, using 30 times the dry weight. The evaporation process was carried out using a rotary evaporator, and fermentation was conducted using *Lactiplantibacillus plantarum*. Non-fermented eggplant peel extracts (EPs) and fermented eggplant peels, referred to as bio-converted eggplant peels (BEPs), were prepared and used for the experiments, respectively. Furthermore, EPs without incubation were labeled as EP-not Inc, while the remaining extracts subjected to incubation were designated as EP-Inc, BEPs 48-h, and BEPs 72-h. In addition, the extraction yield was determined to be 22% relative to the dry weight. These samples were dissolved in cell/tissue culture-grade water and then stored at −20 °C.

### 2.2. Cell Culture

Human dermal fibroblasts (HDFs) (PromoCell (Heidelberg, Germany)) were cultured in 10% fetal bovine serum (FBS)-DMEM (Welgene, Gyeongsan-si, Republic of Korea) in a 5% CO_2_ incubator at 37 °C.

### 2.3. Measurement of Cytotoxicity

Cell proliferation was detected with a cell proliferation kit (MTT) (Roche, Mannheim, Germany). HDFs were seeded on a 96-well plate. EPs were applied to the cells at various concentrations (0, 10, 50, and 100 ppm) and incubated for 24 h. After incubation, the DMEM containing EPs or BEPs was removed and washed with DPBS. Subsequently, the MTT reagent was incubated for 4 h. Following the reaction, 100 µL of solubilization buffer was incubated for 30 min. The absorbance was read using a microplate reader (BioTek Multi-Mode Microplate Reader, Winooski, VT, USA) at 570 nm.

### 2.4. Wound-Healing Analysis

HDFs were seeded in a 60 mm dish and cultured until over 80% confluence was reached. A scratch was then created at the center of the dish using a 200 μL pipette tip. After removing the culture medium, fresh medium was added, and various concentrations of Eps, BEPs (0, 50, and 100 ppm), and H_2_O_2_ (200 μM) were treated for 24 h, and images were obtained using phase-contrast microscopy (Microscope ECLIPSE Ts2, Nikon, Tokyo, Japan) at 0 and 24 h for comparison; % Migration area = (A_0_ − A_n_)/A_0_ × 100 (A_0_: the initial area, A_n_: the remaining area) [38].

### 2.5. Assessment of Intracellular ROS

The fluorescent marker 2′,7′-dichlorodihydrofluorescein diacetate (DCF-DA) (Sig-ma-Aldrich, St. Louis, MO, USA) was used for detecting ROS from the H_2_O_2_. HDFs were seeded on a 96-well black plate and cultured for 24 h. Subsequently, EPs and BEPs were applied to the cells at various concentrations (0, 50, and 100 ppm) and incubated for 24 h. Cells were stained with 20 mM DCF-DA for 45 min. Fluorescence intensity was detected at 485/528 nm using a BioTek Synergy HTX multimode reader (Winooski, VT, USA).

Additionally, fluorescence analysis was performed to observe DCF-DA staining. HDFs were plated on a confocal dish. The cells were treated with EPs and BEPs (0, 50, and 100 ppm) for 24 h. Cells were stained with 10 μM DCF-DA for 15 min for imaging analysis. Each image was visualized using a Nikon Eclipse Ti2 fluorescence live-cell imaging microscope (Nikon, Tokyo, Japan).

### 2.6. Membrane Potential of Mitochondria

The mitochondrial membrane potential was measured with a JC-1 assay kit (Abcam, Cambridge, UK). HDFs were plated on confocal dishes and treated with EPs and BEPs (100 ppm) for 24 h. Cells were washed with DPBS and exposed to H_2_O_2_ (200 μM). Cells were washed again and incubated with JC-1 solution at 5 μM for 15 min. Fluorescence images were acquired using a Nikon Eclipse Ti2 microscope (Nikon, Tokyo, Japan).

### 2.7. Western Blot Analysis

HDFs were seeded in a 100 mm dish and cultured in a CO_2_ incubator. Cells were treated with EPs and BEPs at a concentration of 100 ppm for 24 h, followed by an additional 2 h treatment with H_2_O_2_ (200 μM). Cells were lysed using RIPA buffer containing a phosphatase inhibitor cocktail. The lysates were centrifuged at 12,000, and protein levels were determined by the BCA assay. Proteins (40 μg/μL) were resolved by SDS-PAGE and transferred onto PVDF membranes. Following blocking with 5% skim milk, the membranes were incubated with primary antibodies against *p*-ERK, total ERK, *p*-p38, total p38, p-JNK, and total JNK, followed by secondary antibody incubation at room temperature for 2 h, and were washed with TBS-T. The membranes were reacted with an ECL reagent, and images were captured using the Invitrogen iBright 1500 imaging system (Waltham, MA, USA). The results were analyzed using ImageJ software version 2.9.0 (National Institutes of Health, Bethesda, MD, USA).

### 2.8. In Vivo Antioxidant Effect of BEPs

Male hairless mice (15–20 g, 4 weeks old) were purchased from Samtaco (Osan, Pyeongtaek, Republic of Korea). They were adapted for one week before the experiment. All animal experiments were performed following the standard guidelines of the Institutional Animal Care and Use Committee (IACUC) at Konkuk University (reference number: KU23213). The mice group were divided into six groups (*n* = 7 per group): (1) control (no UVB, 0.9% saline, 200 µL), (2) UVB-only (100–120 mJ/cm^2^), (3) UVB + EPs (200 mg/kg), and (4) UVB + BEPs (200 mg/kg). UV irradiation (Vilber, Collégien, France) was applied every two days (1.5 cm^2^, 100–120 mJ/cm^2^) for approximately 10 sessions until visible wrinkling appeared. Samples (200 µL) were orally administered using a gavage needle 5 days a week for a duration of 4 weeks. Skin wrinkle progression was monitored weekly to determine the experimental endpoint.

### 2.9. Histological Analysis

Dorsal skin tissues were harvested for analysis. The excised tissues were fixed in 10% formalin (HuBENTech, Damyang, Republic of Korea). Hematoxylin and Eosin (H&E), immunohistochemistry (IHC), and Masson’s trichrome (MT) were performed in KP&T Technology Company (Osong, Cheongju-si, Republic of Korea). IHC staining was assessed for the expression of collagen I (COL1) and matrix metalloproteinase 1 (MMP1). Quantification of the MT, COL1, and MMP1-stained tissues was carried out using ImageJ software version 2.9.0

### 2.10. Statistical Analysis

The results are presented as the mean ± standard deviation (SD). Statistical analyses were conducted using GraphPad Prism 5.0 software (San Diego, CA, USA) employing one-way analysis of variance (ANOVA) with Tukey’s Multiple Comparison Test. Statistical significance was considered for *p* values < 0.05.

## 3. Result

### 3.1. Effect of EPs and BEPs on the Viability of HDFs

**Figure 1:**
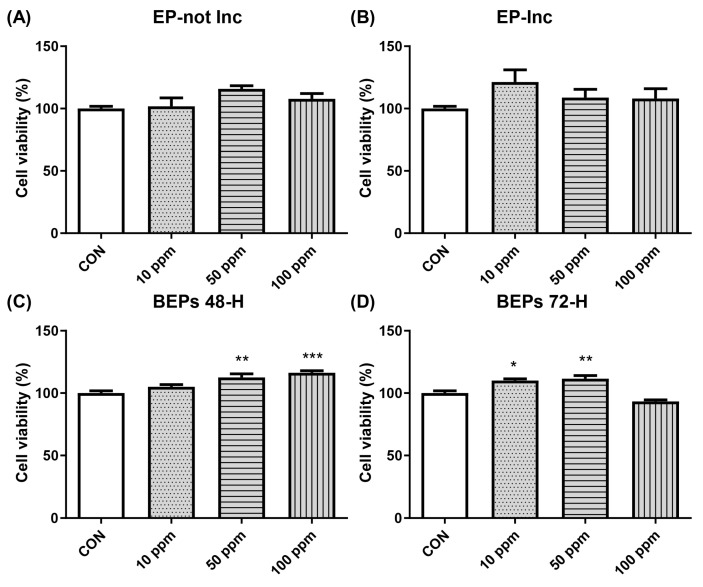
The cell viability of both EPs and BEPs in HDFs. (**A**) EP-not Inc, (**B**) EP-Inc, (**C**) BEPs 48-h, and (**D**) BEPs 72-h at various concentrations were assessed for their effects on cell viability (*n* = 4). Data are presented as mean values ± SD. * *p* < 0.05, ** *p* < 0.01, *** *p* < 0.001, compared to the control group.

The potential cytotoxicity of EPs and BEPs on HDFs was evaluated using the MTT assay. HDFs were administered with each extract at varying concentrations (10, 50, and 100 ppm). Cell viability exceeded 90% across all concentrations, indicating no significant cytotoxic effects of EPs or BEPs on HDFs (Figure 1). Based on these findings, 50 ppm and 100 ppm concentrations of EPs and BEPs were selected for further efficacy analysis.

### 3.2. Wound-Healing Effects of BEPs in H_2_O_2_-Damaged HDFs

**Figure 2:**
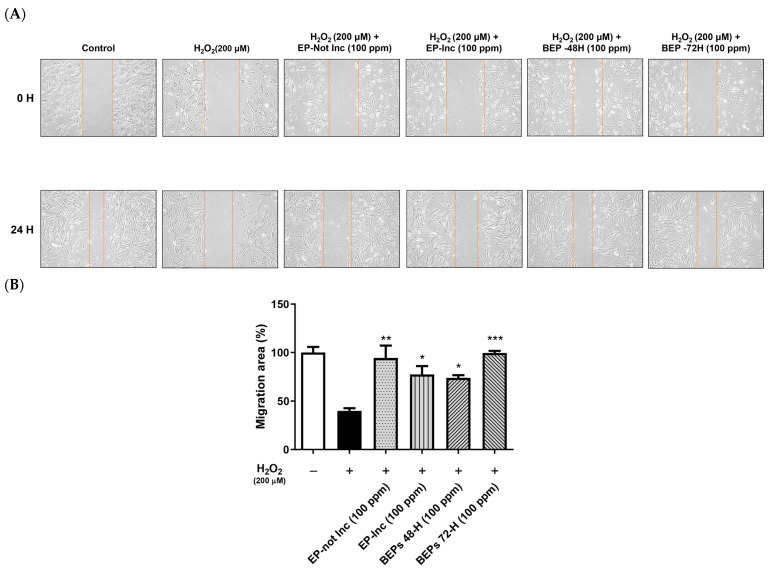
BEPs promoted wound healing in H_2_O_2_-damaged HDFs. (**A**) The wound-healing effects of EPs and BEPs were assessed (*n* = 3). (**B**) The wound closure area corresponding to EP and BEP treatments is presented (*n* = 3). Data are presented as mean values ± SD. * *p* < 0.05, ** *p* < 0.01, *** *p* < 0.001, compared to the H_2_O_2_-damaged group.

The migration capacity of HDFs plays a pivotal role in skin regeneration and wound-healing processes [39]. To evaluate this, a scratch assay was performed to investigate the effects of EPs and BEPs on cell migration in H_2_O_2_-damaged HDFs. HDFs were scratched and treated with H_2_O_2_ (200 μM), followed by the application of EPs and BEPs at concentrations of 100 ppm (Figure 2A), respectively. Wound-healing efficiency was assessed over time. After 24 h, all EPs and BEPs exhibited wound-healing activity, as evidenced by a graphical analysis of cell migration (Figure 2B). Among the treatments, BEPs 72-h demonstrated the highest wound-healing efficacy compared to other samples.

### 3.3. Inhibitory Effect of BEPs on ROS Generation in H_2_O_2_-Damaged HDFs

**Figure 3:**
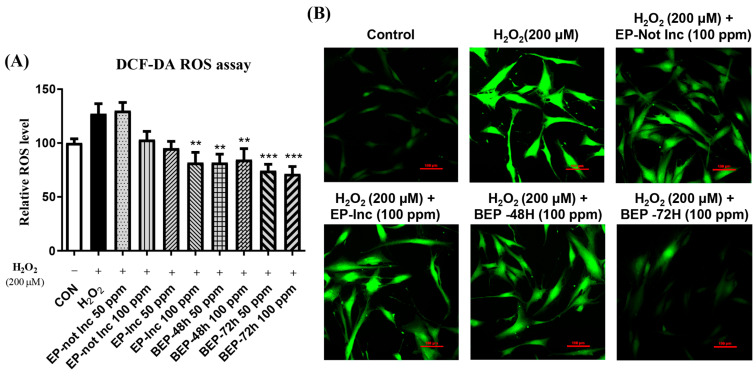
BEPs decreased ROS levels in H_2_O_2_-damaged HDFs. (**A**) The antioxidant capacity of EPs and BEPs (50, 100 ppm) was evaluated using the DCF-DA assay (*n* = 3). (**B**) DCF-DA fluorescence images of EPs and BEPs were captured using fluorescence microscopy (*n* = 3). Data were presented as mean values ± SD. ** *p* < 0.05, *** *p* < 0.001, compared to the H_2_O_2_-damaged group.

Excessive oxidative stress causes cellular damage, particularly in the skin, where it accelerates aging, inflammation, and the formation of wrinkles [40,41]. ROS are byproducts of normal cellular metabolism. However, under stress conditions, their accumulation induces oxidative damage to cellular structures [42,43]. The effects of EPs and BEPs inhibiting ROS production in H_2_O_2_-damaged HDFs were evaluated using a DCF-DA assay. The results demonstrated an elevated level of ROS levels in the H_2_O_2_-damaged group, whereas the BEP group exhibited a marked reduction in ROS levels compared to the EP-not Inc group. Among the treatments, the BEPs 72-h group demonstrated the most effective inhibition of ROS production (Figure 3A). Additionally, DCF-DA fluorescence was observed in HDFs using fluorescence microscopy. The elevated ROS levels in the H_2_O_2_-damaged group were significantly reduced in the BEPs 72-h group (Figure 3B), suggesting that the BEPs 72-h sample possesses higher potential activity in reducing H_2_O_2_-induced ROS compared to EP samples.

### 3.4. Effects of BEPs on the Membrane Potential of Mitochondria in H_2_O_2_-Damaged HDFs

**Figure 4:**
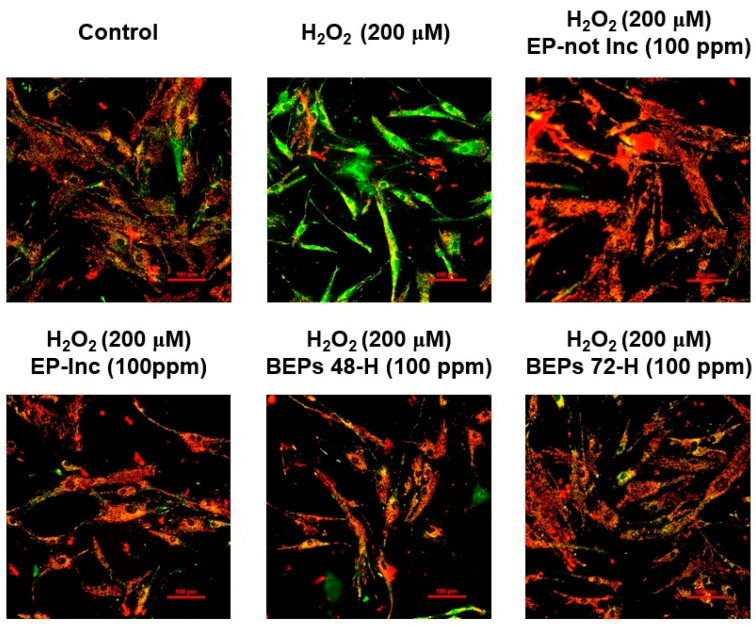
Effects of EPs and BEPs on the mitochondrial membrane potential in H_2_O_2_-damaged HDFs. The membrane potential of mitochondria was assessed using JC-1 in response to treatment with EPs and BEPs (*n* = 3). These images were representative images of three independent experiments (scale bars, 100 µm).

Mitochondria are essential for regulating cellular metabolism and homeostasis by generating ATP and defending against oxidative stress [44,45]. In the skin, mitochondria support critical processes such as aging prevention, wound healing, and cellular repair, thereby preserving structural and functional integrity [46]. Mitochondrial membrane potential (ΔΨm) serves as a vital indicator of mitochondrial health, regulating ATP production and cellular redox balance. Loss of ΔΨm is associated with increased ROS generation and exacerbation of skin damage [47]. The JC-1 mitochondrial membrane potential assay was performed to assess the effects of EPs and BEPs on changes in ΔΨm in H_2_O_2_-damaged HDFs. A polarized mitochondrial membrane potential is signified by the red fluorescence of JC-1 dimers, whereas depolarized membrane potential is represented by the green fluorescence of JC-1 monomers [48]. Treatment with EPs or BEPs at a concentration of 100 ppm in H_2_O_2_-damaged HDFs resulted in a reduction in green fluorescence and an increase in red fluorescence, similar to the control group (Figure 4). These findings suggest that EPs and BEPs effectively restore mitochondrial membrane potential in H_2_O_2_-damaged HDFs.

### 3.5. Effect of BEPs on the Phosphorylation Levels of MAPKs in H_2_O_2_-Damaged HDFs

**Figure 5:**
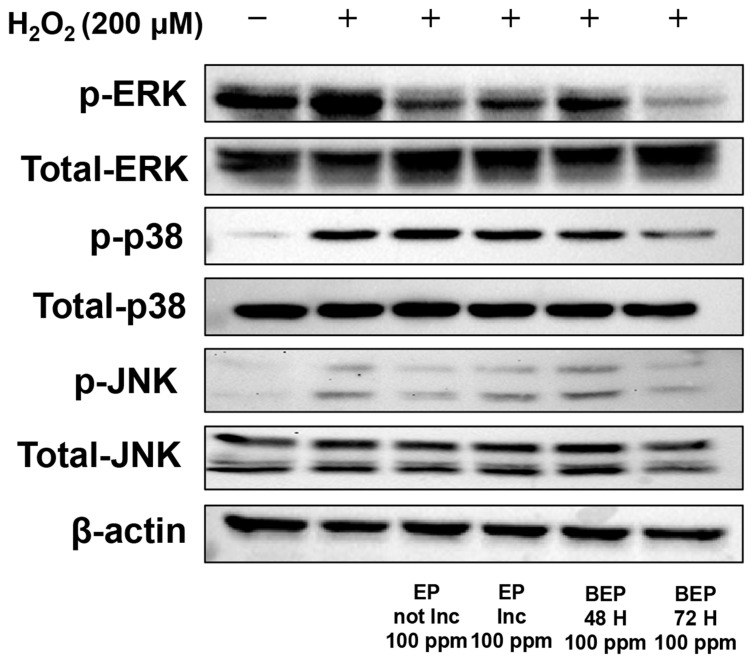
Effect of BEPs on the phosphorylation levels of MAPKs in H_2_O_2_-damaged HDFs. Western blot analysis was performed to assess the inhibitory effects of EPs and BEPs on MAPKs. This image was a representative image from 3 independent experiments.

In the MAP kinase (MAPK) signaling pathway, membrane receptors are activated in response to ROS, leading to the phosphorylation of MAPKs, which triggers inflammatory responses and oxidative stress [49,50]. Changes in MAPK phosphorylation were analyzed using a Western blot analysis. In H_2_O_2_-damaged HDFs, an increase in the phosphorylation levels of ERK, p38, and JNK was observed. Importantly, the BEPs 72-h treatment group exhibited a significant decrease in the phosphorylation levels of ERK, p38, and JNK compared to the EPs-treated group (Figure 5). These findings indicate that BEPs, particularly after fermentation, exhibit superior efficacy in suppressing MAPK phosphorylation.

### 3.6. Effect of BEPs on UVB-Induced Skin Damage

**Figure 6:**
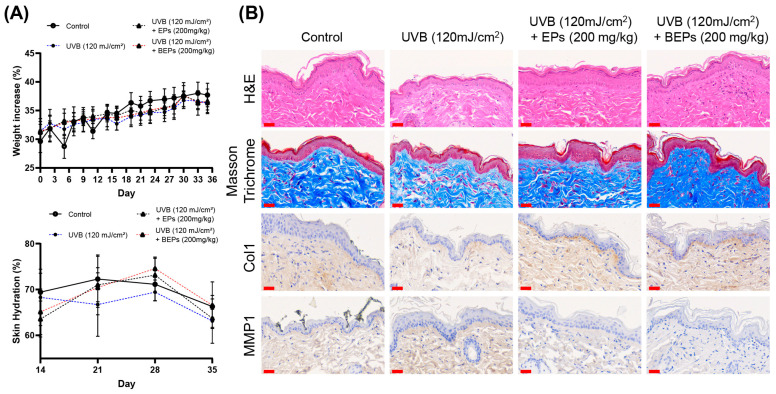
BEPs improve on UVB-induced skin damage. (**A**) Body weights were recorded for each experimental group (*n* = 7). Skin moisture content was assessed on days 14, 21, 28, and 35 during the experimental period (*n* = 7). (**B**) Histological analysis was performed on tissue sections using H&E staining, Masson staining, and immunohistochemical analysis. These images are one of three independent experiments (scale bars, 30 µm).

We further investigated whether EPs and BEPs have beneficial effects on UVB-induced skin damage using hairless mice. All treatment groups, except for the control group, were orally administered the test substances at 200 mg/kg (200 μL) five times per week for three weeks. Skin moisture content was measured weekly throughout the experimental period. Compared to the UVB group, the BEPs group demonstrated significant improvements in skin health, as confirmed by H&E staining (Figure 6). Moisture content measurements further supported these findings, indicating that the fermented extracts contributed to the maintenance of skin hydration. Moreover, the inhibition of MMP1 observed in the fermented extracts group was associated with increased collagen production. Our results suggest that BEPs remarkably mitigate UVB-induced skin damage by improving skin hydration, inhibiting MMP1, and enhancing collagen production, highlighting its potential as a promising agent for improving skin health under UVB-induced stress.

## 4. Discussion

The skin serves as a protective barrier, covering the body’s exterior and playing a vital role in maintaining homeostasis [51]. Beyond its physical barrier function, the skin is critical for thermoregulation, moisture retention, and immune defense, contributing to overall health and functionality. However, the skin is exposed to external factors, such as ultraviolet (UV) radiation, which can induce structural and functional alterations. These changes highlight the significance of understanding the skin’s protective mechanisms to mitigate damage and preserve its integrity [52,53].

UV radiation is known to generate ROS, leading to various detrimental changes in the skin [54,55]. ROS activate MAPKs, which increase MMPs, resulting in the degradation of collagen and subsequent loss of skin elasticity [56,57,58]. Furthermore, mitochondrial dysfunction, a critical regulator of cellular energy metabolism, disrupts energy homeostasis and exacerbates oxidative stress [59,60]. These detrimental effects collectively compromise the structural integrity of the skin.

To counteract the adverse effects of ROS, significant research efforts have been directed toward identifying natural antioxidants. Plant-derived compounds are particularly noteworthy for their potent antioxidant properties [18]. Plants belonging to the *Solanum* genus, such as eggplants, are predominantly found in warm regions worldwide and have been traditionally utilized in both medicine and food applications. These plants are rich in bioactive compounds, including phenols, saponins, flavonoids, and carotenoids, which contribute to their health-promoting properties [61,62]. Eggplants, in particular, are recognized for their high nutritional value and potent antioxidant activity, positioning them as a promising source of antioxidants beneficial for human health [63,64]. These attributes emphasize a potential therapeutic agent for alleviating oxidative stress, reducing inflammation, and promoting skin health.

**Figure 7:**
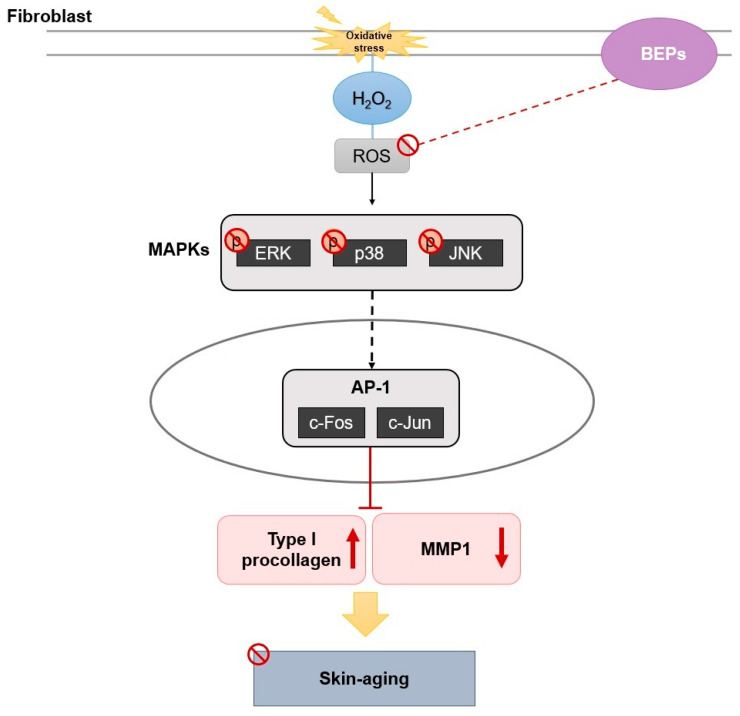
The mechanism of BEPs in UVB-induced skin damage.

In this study, we fermented eggplant peel using *Lactobacillus plantarum* to investigate changes in its efficacy. ROS generation was induced in HDFs through H_2_O_2_ treatment, and the antioxidant potential of fermented BEPs was evaluated. BEPs effectively suppressed ROS production and inhibited the activity of MAPKs. Additionally, BEPs improved mitochondrial dysfunction and demonstrated significant wound-healing effects. In UVB-induced hairless mice models, BEPs reduced MMP1 expression and enhanced collagen production. These findings suggest that BEPs possess superior antioxidant properties compared to non-fermented eggplant peel extracts (EPs) and play a critical role in protecting and improving skin health by promoting collagen synthesis and reducing oxidative stress (Figure 7).

Collectively, the results indicate that fermentation with *Lactobacillus plantarum* enhances the antioxidant and therapeutic properties of eggplant peel, highlighting its potential as a protective and functional food agent against UVB-induced skin damage.

Although the antioxidant and anti-inflammatory properties of BEPs were studied, more research is needed to investigate other biological pathways, including immune modulation and interactions with the gut microbiota. Additionally, the bioavailability and long-term stability of BEPs are not yet fully characterized and require further assessment. Further studies will lead to a deeper understanding of BEPs and broaden their potential applications.

## 5. Conclusions

In conclusion, BEPs effectively reduced ROS levels and the activity of MAPKs, improved mitochondrial dysfunction, and suppressed MMP1 expression while promoting collagen production and wound healing. These results suggest that fermentation with *Lactobacillus plantarum* enhances the antioxidant and protective properties of eggplant peels. Specifically, BEPs demonstrated significant efficacy in mitigating UVB-induced oxidative stress by inhibiting ROS accumulation, downregulating MAPK signaling, and promoting collagen synthesis, thereby contributing to skin protection and repair.

## Data Availability

The original contributions presented in this study are included in the article/Supplementary Materials. Further inquiries can be directed to the corresponding author.

## Supplementary Materials

The following supporting information can be downloaded at: https://www.mdpi.com/article/10.3390/nu17050847/s1.

## Abbreviations

The following abbreviations are used in this manuscript:

BEPs	Bio-converted eggplant peels
COL1	Collagen I
Eps	Eggplant peels
HDFs	Human dermal fibroblasts
MMPs	Matrix metallopeptidase
MAPK	Mitogen-activated protein kinase
UVB	Ultraviolet B
ROS	Reactive oxygen species
H_2_O_2_	Hydrogen peroxide
HDFs	Human dermal fibroblasts
DCFDA	2′, 7′ -dichlorodihydrofluorescein diacetate
ERK	Extracellular signal-regulated kinase
JNK	c-Jun *N*-terminal kinase
FBS	Fetal bovine serum
DMEM	High-glucose Dulbecco’s modified Eagle medium
IHC	Immunohistochemistry
H&E	Hematoxylin and eosin
MT	Masson’s trichrome
CPDs	Cyclobutane pyrimidine dimers
6-4PPs	6-4 photoproducts
