# Supplementary material for: Enhanced Antioxidant and Protective Effects of Fermented Solanum melongena L. Peel Extracts Against Ultraviolet B-Induced Skin Damage
Source: Nutrients. 2025 Feb 28;17(5):847. doi: 10.3390/nu17050847 (PMC11901538; doi:10.3390/nu17050847)

Supplementary figures

Supplementary Figure S1. Flowchart of the study on BEPs

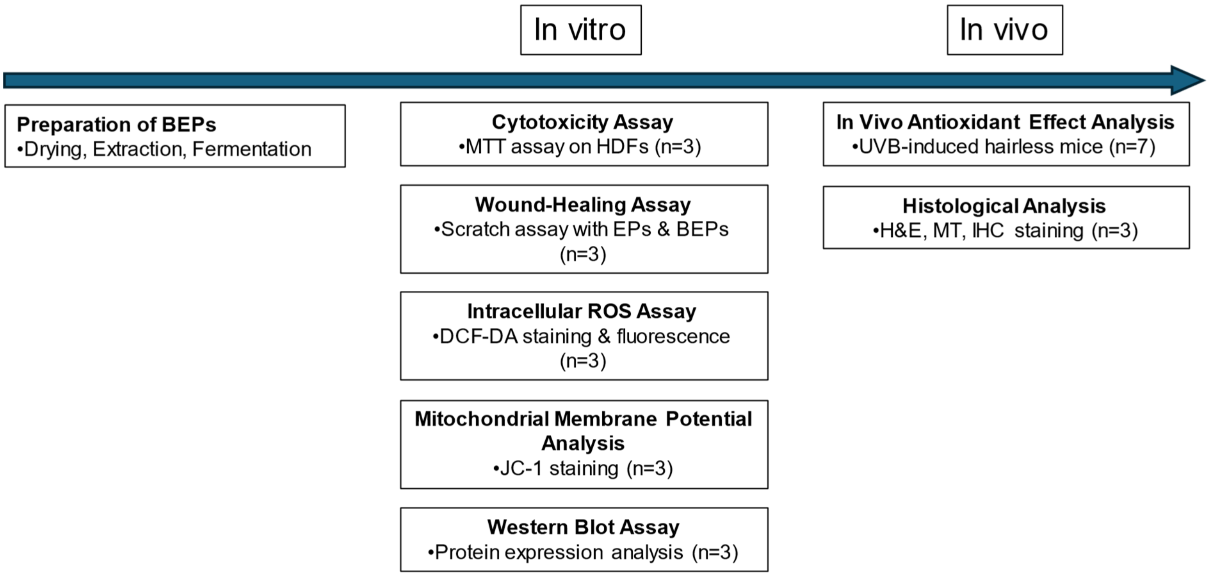

Supplementary Figure S2. Effect of BEPs on the phosphorylation levels of MAPKs in H2O2-damaged HDFs. Western blot analysis was performed to assess the inhibitory effects of EPs and BEPs on MAPKs. This image was a representative image from 3 independent experiments.

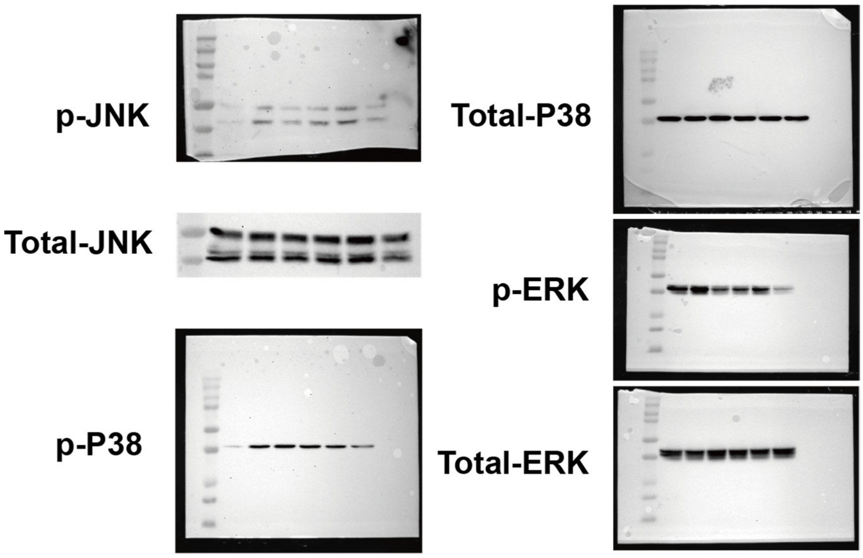

Supplement: Supplementary file 1 [file nutrients-17-00847-s001.zip › nutrients-3472389-supplementary.pdf]
